# 3D kidney organoids for bench-to-bedside translation

**DOI:** 10.1007/s00109-020-01983-y

**Published:** 2020-10-09

**Authors:** Navin Gupta✉, Emre Dilmen, Ryuji Morizane

**Affiliations:** 1 Nephrology Division, Massachusetts General Hospital, Boston, MA USA; 2 Department of Medicine, Harvard Medical School, Boston, MA USA; 3 The Wyss Institute, Harvard University, Cambridge, MA USA; 4 Harvard Stem Cell Institute, Cambridge, MA USA

**Keywords:** Kidney organoid, Stem cells, Bioengineering, Nephron

## Abstract

The kidneys are essential organs that filter the blood, removing urinary waste while maintaining fluid and electrolyte homeostasis. Current conventional research models such as static cell cultures and animal models are insufficient to grasp the complex human in vivo situation or lack translational value. To accelerate kidney research, novel research tools are required. Recent developments have allowed the directed differentiation of induced pluripotent stem cells to generate kidney organoids. Kidney organoids resemble the human kidney in vitro and can be applied in regenerative medicine and as developmental, toxicity, and disease models. Although current studies have shown great promise, challenges remain including the immaturity, limited reproducibility, and lack of perfusable vascular and collecting duct systems. This review gives an overview of our current understanding of nephrogenesis that enabled the generation of kidney organoids. Next, the potential applications of kidney organoids are discussed followed by future perspectives. This review proposes that advancement in kidney organoid research will be facilitated through our increasing knowledge on nephrogenesis and combining promising techniques such as organ-on-a-chip models.

## Introduction

Kidneys are essential organs that filter the blood to generate metabolic waste, destined for urinary excretion. The kidneys have remarkable plasticity in tailoring the composition of urine, matching intake with excretion to maintain solute homeostasis and fluid balance. As the mammalian kidney is a non-regenerative organ, loss of functional units, or nephrons, accumulates over time in all individuals [1]. Systemic diseases of high prevalence, namely, hypertension and diabetes mellitus, medication or hemodynamic-induced acute kidney injury (AKI), and primary diseases of the kidney, are common causes for hastened loss of kidney function and comprise the major etiologies for chronic kidney disease (CKD) [2]. CKD is divided into stages based on kidney dysfunction, which causes solute derangements and fluid imbalance responsible for extensive morbidity and mortality. In the USA, CKD has a reported prevalence of 14% [3], has an annual treatment cost of $120 billion [4], negatively impacts quality of life [5], and at advanced stages culminates in end-stage renal disease (ESRD), which requires the need for renal replacement therapy (RRT). Forms of RRT include dialysis and transplantation, the former accounting for 7% of the Medicare budget [6] and the latter limited by the supply of suitable organs [7]. CKD and ESRD present a major burden on healthcare resources and costs, as well as to patients suffering from these diseases [8].

To address these burdens, fundamental and translational research has been conducted in the fields of kidney development, nephrotoxicity testing, disease modeling, and kidney regenerative medicine. Traditional research tools consist of in vitro cell culture, from both isolated primary kidney cells and virally transformed immortalized cell lines, and in vivo animal models. While these common models have been used to study kidney physiology and pathology with some success, they have inherent limitations that may contribute to the poor translation of lab studies to clinical therapy [9]. In vitro models are largely restricted to analysis of single cell types, ignoring the effects of intercellular and environmental interactions within complex heterogeneous tissue, such as the kidney. Animal models, while integrative of bodily systems, are limited by inter-species differences, resulting in non-faithful conclusions that come at a high expense of time and cost [10]. Novel methods of experimentation in human cells and tissues are a necessity to overcome the limitations of traditional research techniques [11].

As necessity is the mother of innovation, advances in protocols using human pluripotent stem cells (hPSCs) have generated complex three-dimensional, multicellular, and organ-specific tissue, termed organoids. Liver, lung, heart, intestines, and kidney organoids permit experimentation in human tissues in vitro [12]. In theory, tissues that comprise the entirety of the human soma can be reproduced at scale, by virtue of hPSCs defining characteristics of pluripotency and self-renewal. The two types of hPSCs are human embryonic stem cells (ESCs) and human induced pluripotent stem cells (iPSCs). In 1998, Thomson et al. first described the generation of ESC lines derived from human blastocysts. To circumvent controversy related to the embryonic origin of ESCs, in 2006, Takahashi et al. described a revolutionary method to induce pluripotency in terminally differentiated somatic cells. Termed iPSCs, these cells are formed by the transcriptional activation of Yamanaka factors (Oct4, Sox2, Klf4, and c-Myc) [13]. Apart from avoiding ethical issues, iPSCs further permit personalized medicine approaches. iPSCs represent an unlimited supply of cells that are isogenic to their parental somatic cell [13]. Patient-specific iPSCs with naturally occurring mutations may be used to generate clinically relevant disease models in human tissue, particularly in conditions for which no animal model exists. In inheritable disease models, genome-corrected controls may establish a direct link between genotype and phenotype [14]. Immunocompetent regenerative medicine approaches may employ bulk, organ-specific tissue generated from a patient’s iPSCs, negating the need for immunosuppression and bypassing the shortage of transplantable organs [15]. Leveraging the translational utility of hPSCs, particularly iPSCs, may revolutionize the fields of drug development and organ transplantation leading to dramatic outcomes on human health and disease.

The ability of hPSCs to reconstitute all the cells of the body poses a grand challenge, specifically, how to control differentiation towards specific cell and tissue types of interest. Mammalian organogenesis serves as the archetype for directed differentiation, the efficient sequential induction of intermediate cell types which occur during organ development [16]. Conversely, direct reprogramming involves the induction of cell type–specific transcription factors, bypassing intermediate cell types during the conversion of a somatic cell towards a defined target population. While benefiting from simpler methodology, direct reprogramming often results in incomplete conversion to the target cell due to the DNA methylation profile and epigenetics of the parental cell [17]. Even if direct reprogramming occurred with complete conversion, the result is likely to be a uniform cellular population [18]. With regard to the kidney, more than a dozen distinct cell types contribute to the structure and function of the nephron in vivo. The reconstitution of a nephron would necessitate direct reprogramming to a common ancestor of all contributing cell types, namely, nephron progenitor cells, endothelial progenitor cells, and stromal progenitor cells [19]. The required approach to developing tissue akin to the native kidney is directed differentiation, a method predicated upon profound knowledge of kidney development. In this review, we highlight the essential role which kidney developmental biology has played on the generation of hPSC-derived kidney organoids, briefly comment on applications of kidney organoid technology, and discuss future directions that address present limitations and challenges to clinical translation.

### Kidney development

Kidney development serves as the gold standard for directed differentiation, to produce in vitro tissue most biosimilar to its in vivo counterpart. Decades of developmental studies in mammalian models revealed that Wnt, FGF, retinoic acid, and TGF-β/BMP pathways govern morphogenesis, tissue patterning, and the induction of organ primordia [12]. These pathways are critical to the iterative development of tissues across the 3 germ layers, including the mesoderm-derived kidney. The mesoderm, sandwiched between endodermal and ectodermal layers, is patterned by the gastrulation of hPSCs (anterior epiblasts) through the primitive streak, a transient structure that defines the anterior-posterior and medial-lateral axes of the developing embryo [12]. The anterior-posterior axis of the mesoderm is determined by the duration of involution through the primitive streak with temporal exposure to Wnt signaling, as hPSCs that involute through the primitive streak migrate anteriorly to initially contribute to anterior structures, filling posteriorly thereafter [1]. The medial-lateral axis of the mesoderm is determined by a rostrocaudal BMP gradient in the primitive streak, as hPSCs undergoing gastrulation through the rostral primitive streak (low BMP activity) become paraxial mesoderm, through mid-primitive streak (moderate BMP activity) become intermediate mesoderm (IM), and through caudal primitive streak (high BMP activity) become lateral plate mesoderm [20, 21]. In the primitive streak, Wnt signal duration and BMP signal degree are major determinants to patterning throughout the mesodermal germ layer.

**Fig. 1:**
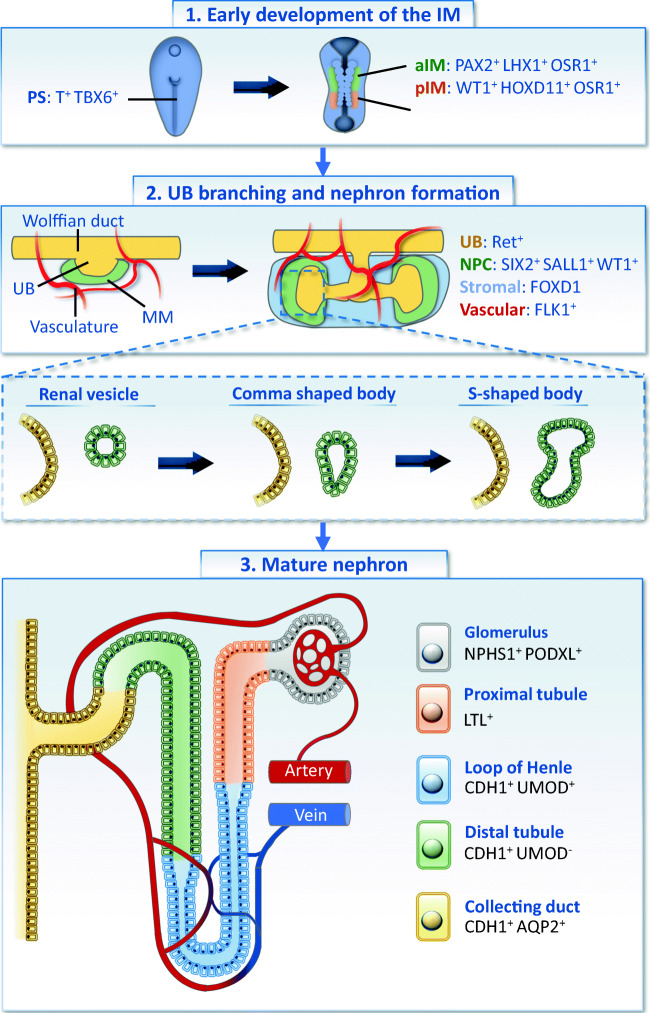
The development of the human kidney from the primitive streak to a mature nephron. [1] The primitive streak (PS) gives rise to the mesoderm and subsequently to intermediate mesoderm (IM) from which posterior IM (pIM) and anterior IM (aIM) can be distinguished during early gastrulation. [2] Metanephric mesenchyme (MM) that includes nephron progenitor cells (NPCs) and stromal cells is formed from the pIM whereas the aIM forms the Wolffian duct from which ureteric bud (UB) develops. The UB starts branching with the NPCs, stromal progenitor cells (SPC), and endothelial progenitor cells (EPC). At this stage, the vasculature does not infiltrate the NPC populations. [3] Next, the NPCs develop renal vesicles that form a comma-shaped body followed by an s-shaped body. Eventually, this structure connects with UB and becomes a mature nephron. The blood is filtered in the glomerulus (gray) which moves the ultrafiltrate to the collecting tubule (yellow) through the proximal tubule (orange), loop of Henle (blue), and the distal tubule (green). Markers corresponding for each structure are given

In mammalian kidney development, there are 3 nephric stages, the pronephros, mesonephros, and metanephros, which all derive from the mesoderm. Studies in mice reveal that the metanephros, which persists as the adult kidney, is derived from the reciprocal induction of two IM-derived tissues, the metanephric mesenchyme (MM) and ureteric bud (UB) [22]. The MM is composed of nephron progenitor cells (NPCs) interspersed with stromal cells, while the UB forms as a remnant outpouching from the Wolffian duct, a degenerative structure that drains the primitive mesonephros to the cloaca [23]. Concentrated NPCs of the MM’s cap mesenchyme undergo progressive morphogenesis from comma-shaped to S-shaped bodies, which segmentally differentiate into contiguous epithelial structures comprising the nephron, namely, podocytes, proximal tubules, loop of Henle, distal tubules, and connecting tubules [24–27]. The connecting tubules of individual nephrons converge in a branched collecting system derived from the UB, following reciprocal induction between the two tissues [28]. UB-derived Wnt signaling catalyzes the mesenchymal-to-epithelial transition (MET) of NPCs to nephron epithelia [29], while MM-derived GDNF signals through the Ret/GFRA1 complex govern branching morphogenesis [30] (Fig. 1).

A major breakthrough in our understanding of kidney development occurred in 2014, when Taguchi et al. unexpectedly discovered that T^+^ MM precursors are developmentally distinct from Osr1^+^ UB progenitors [31], using lineage tracing techniques, noting that prior work suggested common ancestry in Osr1^+^ IM without further delineation [22]. In the aforementioned study, an Osr1-GFP mouse was used to determine that Osr1 is expressed at different times and different locations in the nephric zone of the IM. By histological analysis and sorting/reaggregating OSR1-GFP^+^ cells at different development stages, the authors were able to demonstrate that anterior IM contributes to the mesonephros, mesonephric duct (Wolffian duct), and the ureteric bud (outpouching of the Wolffian duct), whereas the posterior IM contains Six2^+^Sall1^+^ NPCs of the MM that differentiate into the epithelial cells of the nephron. The determination that MM is derived from the posterior IM, and UB from the anterior IM, along with characteristic marker expression, provides a roadmap for the specific induction of the kidney-specific lineages and has enabled directed differentiation protocols to efficiently induce MM-derived nephron epithelia without contamination of other IM derivatives. Importantly, historical studies assumed mouse kidney development reflected human nephrogenesis. Comparing human and mouse kidney development, recent studies have elucidated both convergent and divergent features, including lobe formation, progenitor niche organization, and the expression of presumptive cell type–defining markers [32]. In such studies, human kidney data has been obtained from posthumous embryonic tissue, allowing for insights as opposed to experimentation. The advent of hPSC-derived kidney organoids permits testable hypotheses in human kidney development, among translational applications.

### 3D kidney organoids

Recent developments have enabled the formation of kidney organoids from human-induced pluripotent stem cells (hiPCS) through directed differentiation [33], generating a novel translational platform for drug development and regenerative medicine techniques. Organoids are mini in vitro organs that self-organize from (stem) cells in 3D microenvironments. They exhibit high similarities with the in vivo counterparts in terms of tissue morphology, cell count, proliferation, and differentiation [34].

After delineating the distinct origins of the MM and UB during mammalian kidney development and working backwards in a stepwise approach to identify intermediate stages and their characteristic marker expression, Taguchi et al. established directed differentiation protocols to MM in both mouse and human PSCs [31]. Specifically, embryoid bodies formed from *OCT3/4*^*+*^*SOX2*^*+*^ hPSCs were subject to extended Wnt and BMP4 signaling to induce *T*^*+*^*CDX2*^*+*^ nascent mesoderm, which is destined to contribute to cells of posterior nascent mesoderm. The medial-lateral axis of mesoderm was directed towards WT1^+^HOXD11^+^OSR1^+^ posterior IM by concurrent treatment with BMP4, Activin and retinoic acid. Next, low Wnt and FGF9 was used to support the development of *OSR1*^+^*SIX2*^+^*SALL1*^+^*WT1*^+^ NPCs, with a reported induction efficiency of ~62%. While NPCs were generated from both mouse and human PSCs, coculture with mouse embryonic spinal cord was necessary to further differentiate them into proximal tubules, distal tubules, and podocyte clusters.

Using both hESCs and hiPSCs in monolayer culture, Morizane et al. described a directed differentiation protocol that induced SIX2^+^SALL1^+^WT1^+^ NPCs with up to 90% efficiency by differentiation days 8–9. Briefly, extended Wnt signaling induced late primitive streak which was subject to activin to form pIM, which differentiated into NPCs in response to FGF9. NPCs were then aggregated in three-dimensional suspension culture by centrifugation, subject to a transient CHIR99021 (CHIR) pulse to simulate UB-derived Wnt signaling to catalyze MET in NPCs, and continued to be subject to FGF9 to expand and maintain the stemness of the NPC niche [35]. Following the CHIR pulse, MM transitioned to a *PAX8*^*+*^*LHX1*^*+*^ pretubular aggregate, which further differentiated into a *LAM1*^*+*^ renal vesicle by differentiation day 14. In stochastic differentiation (i.e., no further factors), renal vesicles self-patterned into nephron-like structures consisting of contiguous epithelia that bear multiple markers for each of podocytes (NPHS1^+^PODXL^+^), proximal tubules (LTL^+^), loop of Henle (CDH1^+^UMOD^+^), distal tubules (CDH1^+^UMOD^−^), and connecting tubules (CDH1^+^AQP2^+^) [36].

**Fig. 2:**
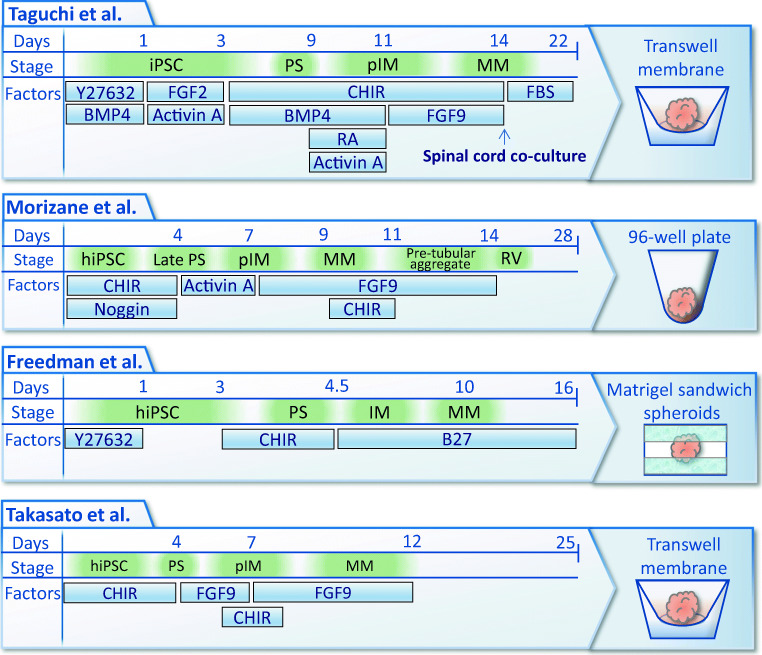
Directed differentiation protocols to generate human-induced pluripotent stem cell–derived kidney organoids. The similarities and the differences between the four protocols to generate human kidney organoids are visualized. Each visualized protocol includes the timescale in days, the stage of the organoid development, and the supplemented factors to drive the directed differentiation and the maturation of the organoids. The protocols of Taguchi et al. and Takasato et al. resulted in organoids on Transwell membranes. Morizane et al. enabled the generation of the kidney organoids in 96-well plates and Freedman et al. in a Matrigel sandwich approach

Freedman et al. have used a different approach to create a simplified protocol to obtain kidney organoids. Here, the authors have used a sandwich Matrigel method to provide a 3D microenvironment for hPSCs to form spheroids surrounding hollow, amniotic-like cavities [37]. Through directed differentiation, these spheroids are stimulated to become kidney organoids with a two-step protocol. The formed spheroids were treated with CHIR for 1.5 days followed by exposure to B27-supplemented media. On day 10, convoluted, translucent, tubular organoids were formed after mesenchymal-to-epithelial transition. Takasato et al. have identified developmental features of both the collecting duct and the kidney mesenchyme progenitors [38]. Here, the authors used this information to induce both metanephric (vs mesonephric) mesenchyme and uretic epithelium by 4 days of CHIR exposure followed by 3 days of FGF9 exposure. The aggregates were then cultured for 20 days, allowing organoids to form that were corresponding with a first trimester kidney. The authors have claimed the presence of the collecting duct by the presence of GATA3 and ECAD markers. However, this was later evaluated as unreliable as distal and collecting tubules derived from the MM can express these markers as well [39, 40] (Fig. 2).

After the above four directed differentiation protocols were published by 2015, multiple groups have followed a similar approach to sequentially convert hPSCs to late primitive streak, pIM, MM, pretubular aggregate, renal vesicle, and ultimately kidney organoids [41–44]. Wu et al. performed a single-cell transcriptomic as a comparative analysis between the Morizane and Takasato protocols, both against each other and against human fetal kidney (16 weeks gestation) and adult human kidney (62-year-old man with normal kidney function). When evaluated under static conditions at differentiation day 26, both protocols generated immature tissue, each expressing ~ 20% of adult proximal tubule and podocyte transcription factors [41]. However, Tran et al. employed single-cell transcriptomics on kidney organoids developed using the Morizane protocol to support the application of organoid-derived podocytes for disease modeling and the restoration of filtrative function. Podocytes from organoids shared highly similar, progressive transcriptional profiles with human fetal kidneys and recruited host vasculature on transplantation to foster further maturation [42]. Garreta et al. found that the transcriptional profile of kidney organoids was extended to that of second trimester human fetal kidneys by day 16 of differentiation by employing a soft hydrogel to lengthen the duration of a 3D microenvironment to facilitate cell-cell and cell-matrix interactions [44]. Following a similar differentiation protocol, Low et al. performed single-cell RNA sequencing (scRNAseq) that identified a subset of nephron progenitor cells as a potential source of renal vasculature, which differs from studies in mouse development demonstrating the source as FLK1^+^ endothelial progenitor cells, which are distinct from NPCs of the MM [45] and in part derive from FOXD1 stromal progenitor cells [46]. Yet, other major findings included that Wnt signaling governs vascularization and the proportion of proximal versus distal nephron segments, functional maturation of organoids after implantation, and modeling autosomal recessive polycystic kidney disease (ARPKD) using patient-derived iPSCs [43].

### Translational utility of kidney organoids

In general, current research models to study disease or compounds are often limited to cell studies and animal models. Cell models are easy to use and can be applied in high numbers. However, these often include single-cell lines and cannot grasp the complexity of the human in vivo kidney such as the 3D microenvironment and multiple cells present. Therefore, cell models are unable to mimic the in vivo 3D tissue morphology and the heterogeneous and complex microenvironment. To account for the complex in vivo environment, animal models such as mice have been a conventional research model. However, these models are poorly translatable into human conditions [47, 48]. Also, ethical concerns for animal use for research purposes have been increasing [49, 50]. Therefore, novel models that can mimic specifically the human in vivo situation more accurately are required [51].

Organoids have the potential to overcome these limitations due to their unique ability to mimic the in vivo situation of humans. And because organoids are derived from hiPSCs, they can also be used for personalized medicine. For example, certain (combinations of) compounds can be tested with the organoids derived from cells of a specific person to test for toxicity or efficacy. Also, larger-scale applications are possible. For example, organoids can be used as pre-clinical models to determine the characteristics of certain compounds. Additionally, reliable human disease models can be used as clinical trials to accelerate development of treatments for especially rare diseases for which patients are scarce.

**Fig. 3:**
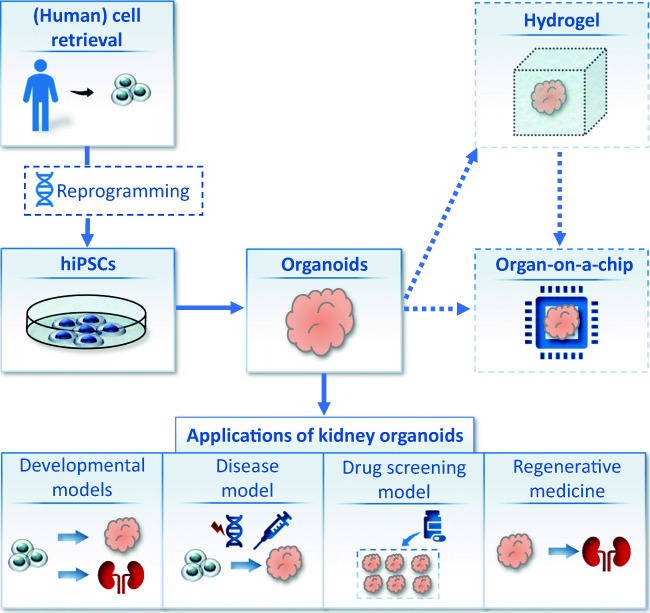
Application of kidney organoids: towards a bench-to-bedside translation. Human-induced pluripotent stem cells (hiPSCs) can be derived from humans to create (patient specific) kidney organoids. In this process, it is also possible to introduce a genetic modification as disease model or to test genetic therapies. The obtained kidney organoids can be placed in hydrogels and/or organ-on-a-chip platforms in order to create higher resemblance to the in vivo situation. The kidney organoids can be applied as developmental models to unveil novel insights in nephrogenesis and subsequently enable the generation of improved kidney organoid models. Disease models can be created by either genetic modification or by exposure to certain compounds known to induce disease. These disease models can also be used in efforts to create novel therapies. Kidney organoids also provide a valuable platform to perform drug screening and toxicity test in large scales. Finally, kidney organoids can contribute to the ultimate goal of recreating a functioning kidney de novo in regenerative medicine

Although more advancements are required to create fully functioning kidneys in vitro, the studies to date show great promise. Some studies have already shown applications of the kidney organoids as they are today. These advancements in kidney organoids enable the (future) application for regenerative medicine and as developmental, toxicity, and disease models. Among these translational applications, organoids are also considered as potential methods for the application for regenerative medicine to create bioengineered kidneys to introduce alternative therapies to dialysis and kidney transplant (Fig. 3). As numerous reviews have reported the published translational applications of kidney organoids [1, 52–59], this review focuses on the future generation of stem cell–derived kidney tissue that is histologically biosimilar to the native human kidney to facilitate translation of kidney organoid research towards impacting clinical care.

### Current challenges and future perspectives

The kidney is a highly vascularized organ that collectively receives 20% of the cardiac output, filtering up to 180 l of blood per day [60]. Nineteen kilometers of glomerular capillaries, with a surface area of ~ 6000 cm^2^, contributes to the glomerular filtration barrier (GFB) [61]. Constituents of the GFB are fenestrated glomerular capillaries, the negatively charged glomerular basement membrane, and podocytes exhibiting slit diaphragms between foot processes [61]. The structure of the GFB begets its function, blood filtration based upon the size and charge of solutes. The primitive urine formed from glomerular filtration enters nephron tubules, where it is processed by absorptive and secretory mechanisms. Of the up to 180 l of filtrate, ~ 99% is absorbed with only 1–2 l destined for urinary excretion under normal conditions. Given substantial volume resorption in peritubular capillaries, it is unsurprising that multiple nephrons drain to shared collecting ducts that converge at the renal pelvis to form solitary ureters. The overall structure, from extensive proximal nephron vascularization to widespread tubular epithelial-peritubular capillary bed communication to arborized distal nephron drainage, plays a critical role in kidney function. Kidneys maintain a relatively necessary function even when capacity decreases to 20%, with many of these patients remaining asymptomatic. However, reductions to below 15% often translate to the need for RRT [62, 63]. NPCs with regenerative abilities are lost in humans before birth, and therefore, the nephrons cannot renew. Currently, there are no therapies available to replenish lost kidney function, so there is high demand for regenerative medicine applications. Moreover, a GFB is required to model diseases that affect filtration, functional tubular transport necessary for a wide variety of toxicity testing and disease modeling, and the combination of MM-derived nephrons with UB-derived collecting ducts necessary to model most congenital anomalies of the kidney and urinary tract (CAKUT) [64]. The development of organized renal vasculature and a branched collecting system would represent significant translational leaps in kidney organoid technology.

### Kidney organoid vascularization

Vascularization of the kidney involves two distinct processes: vasculogenesis and angiogenesis [65]. In vasculogenesis, vasculature assemble occurs de novo, whereas angiogenesis involves the branching or extension of pre-existing vessels. The interplay between these two processes during nephrogenesis remains unknown, yet vasculogenic kidney microvessels may precede the development of the angiogenic renal artery [65]. In mice, the MM has no vasculature during uretic bud invasion but develops a rich capillary network by the following day with the subsequent formation of vascularized glomeruli. The latter is realized by endothelial cells that invade the vascular cleft to form single-capillary loop stage glomeruli [66–68]. Meanwhile, vasculogenic FLK1^+^ endothelial progenitor cells interspersed in the MM contribute to the rich glomerular capillary network [69]. Similar to directed differentiation approaches to forming MM ex vivo, vascularization of hPSC-derived kidney tissue should follow its embryonic origins to develop the patterned, hierarchical renal vascular network.

3D kidney organoids developed from the initial protocols for pIM-derived MM were found to contain vascular cells; however, glomeruli remained largely avascular, and the overall abundance of blood vessels was poor when compared with native kidney tissue [70]. Importantly, these vasculogenic blood vessel assemblies mimic early microvessel formation in developing kidney tissue, occurring prior to angiogenic renal artery invasion [65]. In their seminal work, Taguchi et al. transplanted their induced mouse MM into mice, with angiogenic host-derived vasculature resulting in increased glomerular capillaries. WT1^+^ podocyte clusters co-localized with PECAM1^+^ vessels, which were found to contain host red blood cells on imaging [31]. Similar subcapsular transplantation of kidney organoids has been repeated, demonstrating vasculature within the glomeruli of organoids persistently derives from the host and the formation of an early glomerular basement membrane consisting of fenestrated endothelial cells and podocyte foot processes [71]. A small degree of glomerular filtration has been claimed on subcutaneous transplantation of kidney organoids, based upon intracellular detection in tubules of systemically administered fluorescent dextrans taken to signify uptake from the glomerular filtrate [72]; however, apical uptake enabled by lack of tight junctions throughout Bowman’s capsule to the proximal tubule or basolateral uptake via tubular phagocytic mechanisms cannot be excluded [73]. Building upon their work in which kidney organoids transplanted under the kidney capsule become vascularized by the host and demonstrate glomerular and tubular maturation [71], recently, van den Berg et al. developed an elegant in vivo system employing high-resolution multiphoton imaging to demonstrate size-selective glomerular sieving [74]. Notably, vascularized glomeruli formed from inter-species chimeric tissue fail to overcome limitations related to the ongoing dependence on animal systems, including developmental and physiological differences between species, a significant loss to personalized medicine approaches, low throughput, and high cost.

Kidney organoid vascularization has been achieved in vitro, towards similar results as reported in transplantation experiments. By marrying kidney organoid and kidney-on-a-chip technologies to recapitulate the fluidic microenvironment in vivo, Homan and Gupta et al. found that fluidic shear stress applied to embedded kidney organoids on chip facilitated the formation of perfusable vascular networks developed from the expansion and differentiation of co-induced endothelial progenitor cells. Additionally, the authors demonstrated increased polarity and maturation of tubular epithelial cells and podocytes, which manifest foot processes that abutted glomerular capillaries to form a primitive GBM [70, 75]. Although the kidney organoids were superfused, as opposed to flow limited to within vasculature, the application of fluidic shear stress on whole organoids simulates the interstitial flow generated from compartmental fluid shifts during reabsorption of ~ 99% of the glomerular filtrate. In other words, ~ 99% of the volume in tubular lumens is transmitted across the interstitium and reabsorbed by the peritubular capillaries in vivo. While perfusable vasculature was demonstrated using fluorescent probes, glomerular filtration was not supported by the detection of fluorescence in tubular lumens due to the use of beads that were 500 nm in diameter, while noting the average diameter of fenestrations in glomerular capillaries is ~ 70 nm [76]. Notably, all vasculature in the system derived from the kidney organoids without angiogenic invasion.

Methods to overcome the present challenges include coupling angiogenic vascular networks in vitro with vasculogenic kidney organoids to permit maturation of the GBM with functional glomerular filtration. There are a multitude of plausible reasons for which definitive glomerular filtration has not yet occurred in kidney organoid-on-chip approaches, including lack of longitudinal experimentation, limited hydrostatic pressure transmitted in glomerular capillaries, immaturity of nephron epithelia, and the potential for essential circulating factors facilitating embryonic kidney development. Directly perfusable angiogenic vascular networks in vitro have previously been described, which are capable of supporting thick tissue or invading embedded cellular constructs on chip [77, 78]. Such vasculature may anastomose with organoid-derived microvessels to limit perfusion to the vascular compartment, enabling the transmission of substantial hydrostatic pressure to glomerular capillaries. Other limitations may be addressed by employing longitudinal studies, perfusing with embryonic serum, and using optimal differentiation day organoids (influencing maturity) in such devices. If the angiogenic vascular network was composed of human cells, then limitations related to inter-species differences would be circumvented. Similarly, the use of hPSC-derived blood vessels, as has been described [79], would facilitate personalized medicine approaches for both drug development and regenerative medicine strategies.

### Ureteric bud formation

The production of glomerular ultrafiltrate ex vivo would represent a landmark discovery to facilitate translational applications of stem cell–derived kidney tissue, yet a collecting system would be required for subsequent drainage. A dichotomously branched collecting duct network converges the output of an individual kidney’s ~ 1 million nephrons to a single ureter [60]. The collecting ducts not only transport the filtrate but are also involved in water reabsorption and electrolyte balance via various transporters and channels in its various types of tubular epithelial cells. The collecting ducts are a product of branching morphogenesis of the ureteric bud which is controlled by various molecular signals which can be exploited in vitro to mimic this process, including FGF, GDNF/RET, and Wnt signaling pathways [68].

Kidney developmental studies using microdissected mouse MM and UB provide significant insight into the early formation of what will constitute the adult human kidney. Uninduced MM, separated from epithelial structures of the UB, prevents the maturation of either tissue. However, coculture of MM and UB ex vivo is able to reconstitute the mouse kidney, suggesting that these kidney precursor populations possess a self-autonomous program sufficient to drive kidney organogenesis [80]. From a developmental perspective, the optimal method to develop functional kidney tissue from hPSCs in vitro is likely to involve separately inducing MM and UB with high efficiency and coculture of the two distinct populations.

The previously described differentiation protocols predominantly generate MM, destined to become nephron structures. Directed differentiation towards collecting duct progenitor cells of the UB was first published in 2013, prior to Taguchi delineating the divergent IM origins of MM and UB. Xia et al. converted hPSCs to mesodermal committed tissue using FGF2 and BMP4 for 2 days, then induced UB progenitor cells using a combination of activin, BMP2, and retinoic acid for 2 additional days in a two-step differentiation protocol [81, 82]. hPSC-derived UB progenitor cells self-assembled into inter-species chimeric tissue when combined with embryonic mouse MM. In 2017, by analyzing gene expression profiles during ureteric bud development in mouse embryos, Taguchi et al. have established a protocol to include both MM and UB tissue into the kidney organoids using mouse ESCs (mESCs) [83]. This protocol was predicated upon FACS-sorting CXCR4^+^KIT^+^ Wolffian duct progenitors. Coculture of Wolffian duct progenitors with mESC-derived MM required the presence of stromal progenitor cells to form reassembled organoids that underwent higher-order organogenesis to form nephrons interconnected by a ramified ureteric epithelium. Ultimately, the findings were not replicated in hPSCs-derived MM and UB due to premature cessation of UB branching, which the authors believed may be due to the lack of the stromal progenitor pool [83]. The establishment of directed differentiation to human kidney stromal progenitors and increased access to primary human fetal kidney tissues may discern the cause of the reported incomplete branching morphogenesis. Yet, there may be limitations in the induction efficiency of the requisite cell type, namely, proliferating Ret^+^ UB tip cells, which are responsible for branching and forming a structural linkage with the connecting tubules of distal nephrons [84].

There is a paucity of directed differentiation protocols of hPSCs towards UB and its derivatives, with the published protocols demonstrating limitations. Improved protocols are required to permit the addition of a collecting duct network to span the entirety of nephron-containing kidney organoids. Analogous to maximizing induction efficiency for NPCs based on SIX2 expression, maximizing the induction efficiency for UB tip cells based on Ret expression is one such strategy. Towards this end, an understanding of kidney development is required. Notably, the UB forms as an outpouching of the Wolffian duct (aka mesonephric duct), which is generated from the caudal migration of anterior IM cells down the urogenital sinus to the cloaca, noting these cells undergo MET along the way. The circuitous origins of the UB pose a challenge to the establishment of a robust directed differentiation protocol. However, Ret-expressing mesenchymal cells present in the aIM are capable of contributing to the UB [84], such that a 3-step protocol may convert hPSCs to early primitive streak (step 1), then aIM (step 2), then Ret^+^ UB tip cells (step 3). Notably, the cellular repository of the NIH’s ReBuilding a Kidney (RBK) consortium includes a Ret reporter line (https://www.rebuildingakidney.org/cell-lines/).

## Conclusion

The kidney is a remarkable organ that among other things regulates the body’s water and electrolyte homeostasis. A dysregulation of these processes can lead to serious health complications with high societal and economic burden. Through our understanding of human nephrogenesis in vivo, we shed light on recapitulating this process in vitro. Current advancements have enabled the generation of hiPSC-derived kidney organoids. These kidney organoids resemble the human kidney and provide additional advantages compared with conventional research models. The application of kidney organoids include regenerative medicine and as developmental, toxicity, and disease models. Despite advancements in recent years, kidney organoids require improved protocols and modifications towards tissue maturation, perfusable vasculature, and collecting system integration. Next, these systems need to be fit to provide additional value to the conventional cell and animal research models or even replace them. Improving the organoid generation protocols through our increasing knowledge of kidney development, combined with the embracement of organ-on-a-chip technology, may facilitate and accelerate the translation of 3D kidney organoids towards clinical use.
